# The Western Japan Atopic Dermatitis Registry (WJADR): A Multicenter Real-World Registry of Systemic Therapies for Atopic Dermatitis

**DOI:** 10.3390/jcm15135232

**Published:** 2026-07-04

**Authors:** Kazuhiko Yamamura, Shu Yotsumoto, Emi Sato, Sakae Kaneko, Yutaka Hatano, Shinichi Imafuku, Takeshi Nakahara

**Affiliations:** 1 Department of Dermatology, Graduate School of Medical Sciences, Kyushu University, Fukuoka 812-8582, Japan; 2 Department of Dermatology, Fukuoka University Faculty of Medicine, Fukuoka 814-0180, Japan; 3 Department of Dermatology, Masuda Red Cross Hospital, Masuda 698-8501, Japan; 4 Department of Dermatology, Faculty of Medicine, Oita University, Oita 879-5593, Japan

**Keywords:** atopic dermatitis, registry, real-world evidence, drug survival, biologics, JAK inhibitors, precision medicine, phenotype, WJADR

## Abstract

**Background**: Atopic dermatitis (AD) is a chronic inflammatory skin disease with substantial impact on quality of life. The introduction of biologics and Janus kinase (JAK) inhibitors has markedly transformed systemic treatment strategies. However, long-term prospective real-world registries evaluating drug survival, safety, phenotype-specific treatment response, and post-discontinuation outcomes remain limited, particularly in Asian populations. **Methods**: The Western Japan Atopic Dermatitis Registry (WJADR) is a multicenter, prospective, observational registry coordinated by Kyushu University and collaborating institutions across western Japan. Patients initiating or currently receiving systemic therapy for AD are enrolled. Longitudinal data collection includes clinical phenotype classification, disease course classification, treatment exposure, physician-assessed severity scores, patient-reported outcomes, biomarkers, and safety information. The primary outcome is drug survival, while secondary outcomes include clinical improvement, adverse events, phenotype–treatment interactions, biomarker–treatment correlations, and treatment-switch patterns. **Results**: WJADR was designed as a phenotype-integrated real-world registry to evaluate comprehensive systemic treatment strategies and post-discontinuation outcomes in AD prior to the completion of patient enrollment and outcome analyses. Unlike existing registries primarily focused on biologic initiator cohorts or treatment burden, WJADR integrates clinical phenotypes, biomarkers, and longitudinal outcomes to support precision medicine approaches. **Conclusions**: WJADR represents the first large-scale multicenter prospective AD registry in western Japan and may provide ethnicity-specific real-world evidence to support long-term safety evaluation, treatment optimization, and phenotype-guided therapeutic strategies in AD.

## 1. Introduction

Atopic dermatitis (AD) is a chronic relapsing inflammatory skin disorder affecting both children and adults and significantly impairing quality of life [1,2]. Persistent pruritus and visible skin lesions contribute to psychosocial burden and mental health comorbidities [2]. Before the advent of targeted therapies, systemic treatment options for AD were limited to conventional immunosuppressive agents, including cyclosporine, methotrexate, azathioprine, and mycophenolate mofetil, many of which were associated with safety concerns and variable long-term effectiveness [3]. Recent international and Japanese guidelines have also established biologics and JAK inhibitors as key systemic therapeutic options for moderate-to-severe AD [4,5]. Phase III trials have demonstrated the efficacy of dupilumab [6], followed by JAK inhibitors including upadacitinib, abrocitinib, and baricitinib [7,8,9], as well as IL-13–targeted monoclonal antibodies [10,11] and IL-31 receptor A-targeting monoclonal antibody [12]. These agents provide distinct mechanisms of action and broaden therapeutic options for moderate-to-severe AD [13,14]. As the therapeutic landscape of AD rapidly evolves, optimization of treatment selection and long-term management strategies has become increasingly important in real-world clinical practice [15,16,17].

Despite robust randomized controlled trial (RCT) evidence, long-term real-world effectiveness, drug survival, switching patterns, and long-term safety profiles, including serious infections and treatment-limiting adverse events, remain incompletely characterized. The importance of real-world evidence (RWE) in guiding therapeutic decision-making has increasingly been recognized [18]. In Europe, the TREatment of ATopic eczema (TREAT) registry established standardized core datasets and safety frameworks and continues to expand harmonized multinational real-world data collection across Europe [18,19,20,21]. Unlike conventional observational cohorts, the TREAT initiative was specifically designed to evaluate long-term safety outcomes, including malignancies, serious infections, and adverse events of special interest, across multiple national registries [20]. Recent pooled analyses from multiple TREAT registries further highlighted the importance of harmonized longitudinal registry data in evaluating treatment outcomes across heterogeneous AD populations [22]. In Japan, the ADDRESS-J registry has provided important real-world data regarding disease burden, patient-reported outcomes, and treatment patterns in Japanese patients with moderate-to-severe AD [23]. However, detailed phenotype-guided analyses, drug survival evaluation, and structured post-discontinuation follow-up were not the primary focus of the registry. In parallel, biologic-focused real-world cohorts such as BioDay, PROSE, GLOBOSTAD, and BIOREP have recently provided valuable longitudinal effectiveness and safety data, particularly for dupilumab-treated patients [24,25,26,27]. Nevertheless, many of these studies primarily evaluated biologic initiator cohorts rather than comprehensive systemic therapy registries designed to compare diverse treatment strategies across heterogeneous AD populations.

AD is increasingly recognized as a highly heterogeneous inflammatory disease rather than a single clinical entity [28]. This heterogeneity may contribute to substantial variability in treatment response and long-term treatment persistence. In addition to classical distribution patterns, AD includes multiple clinical phenotypes such as head-and-neck predominant type, prurigo type, and erythrodermic type [29]. Emerging evidence also suggests that these phenotypes may differ in immunological characteristics, therapeutic responsiveness, and treatment persistence [30]. Consequently, future AD management may require phenotype-guided treatment optimization and precision medicine approaches rather than a “one-size-fits-all” strategy. To address these unmet needs, we established the Western Japan Atopic Dermatitis Registry (WJADR), a multicenter prospective observational registry designed to evaluate long-term safety, effectiveness, drug survival, phenotype–treatment interactions, and post-discontinuation outcomes in real-world AD practice. By integrating clinical phenotypes, disease course classifications, biomarkers, treatment exposure, and longitudinal outcomes, WJADR aims to provide a comprehensive real-world evidence platform for precision medicine in AD. The present article describes the rationale, study design, and methodological framework of the WJADR prior to the completion of patient enrollment and outcome analyses.

## 2. Materials and Methods

### 2.1. Study Design and Setting

The Western Japan Atopic Dermatitis Registry (WJADR) is a multicenter, prospective, observational registry study coordinated by the Department of Dermatology, Kyushu University, in collaboration with institutions across western Japan. The registry is designed to evaluate the long-term effectiveness, safety, drug survival, treatment-switch patterns, and post-discontinuation outcomes of systemic therapies for atopic dermatitis (AD) in routine clinical practice. Patient enrollment begins in April 2026, and additional participating institutions may be incorporated during the study period. The planned target enrollment is approximately 450 patients across participating institutions. This target was determined based on the anticipated number of eligible patients at participating centers and the expected expansion of participating institutions during the study period.

**Figure 1:**
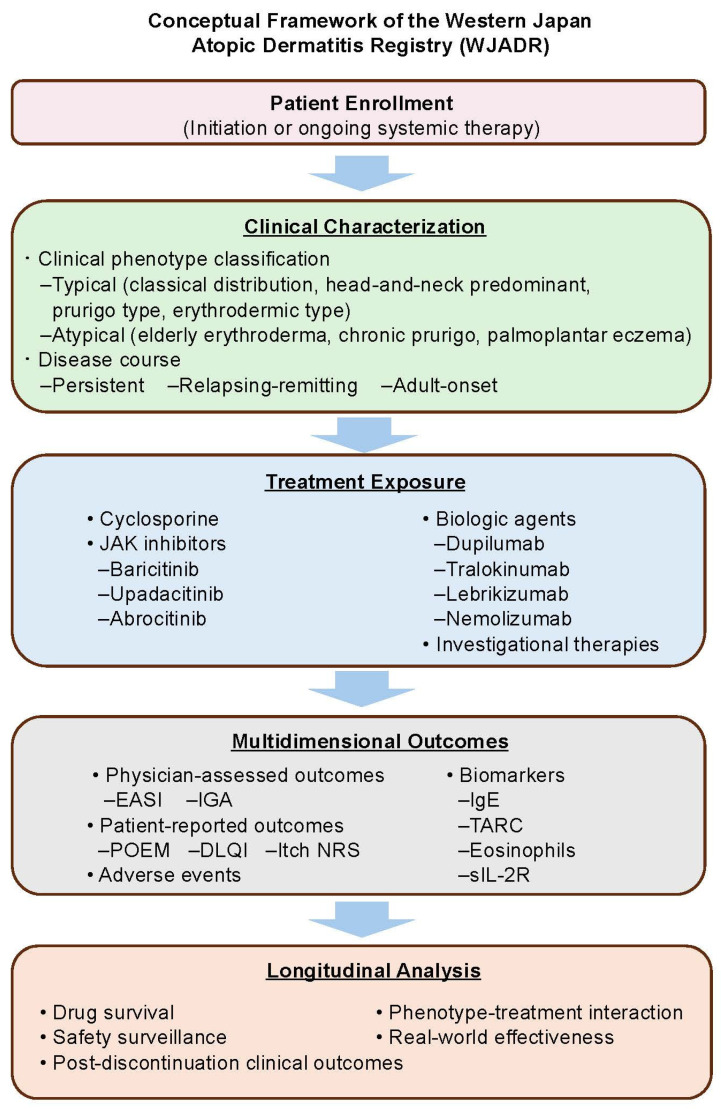
Schematic overview of the design and longitudinal structure of the Western Japan Atopic Dermatitis Registry (WJADR). Patients initiating or currently receiving systemic therapy for atopic dermatitis are enrolled in a multicenter, prospective observational registry. Baseline assessments include demographic data, clinical phenotype classification, disease course, physician-assessed severity, patient-reported outcomes, biomarkers, and safety screening. Follow-up assessments are conducted at 1 month and every 3 months thereafter. The registry captures treatment exposure, drug survival, clinical response, adverse events, and treatment-switch patterns as well as post-discontinuation clinical outcomes, including time to relapse, duration of remission, and treatment re-initiation, to generate real-world evidence in the era of targeted systemic therapies.

As illustrated in Figure 1, clinical information, physician-assessed severity scores, patient-reported outcomes, laboratory biomarkers, treatment exposure, adverse events, and longitudinal follow-up data are collected from participating institutions and integrated into a centralized electronic database system. The registry framework is designed to support phenotype-integrated analyses and precision medicine approaches in AD by incorporating clinical phenotypes, disease course classifications, biomarker profiles, and longitudinal treatment outcomes within a real-world setting. Clinical data collected at each participating institution are anonymized using personal identification codes before transfer to the coordinating center. Longitudinal registry data are securely managed and shared among participating institutions for collaborative analyses.

ChatGPT-5.5 was used as a generative artificial intelligence tool solely for superficial text editing, such as improving grammar and readability.

### 2.2. Patient Population and Eligibility Criteria

Patients diagnosed with AD by board-certified dermatologists and initiating or currently receiving systemic therapy are eligible for enrollment. Systemic therapies include cyclosporine, biologic agents, Janus kinase (JAK) inhibitors, and investigational therapies. Short-term systemic corticosteroids and antihistamines are not classified as systemic therapies in this registry. Concomitant phototherapy is recorded as treatment exposure.

Patients are excluded if the diagnosis of AD is considered uncertain, informed consent cannot be obtained, or the investigators judge the patient to be inappropriate for enrollment.

### 2.3. Clinical Phenotype and Disease Course Classification

Clinical phenotype classification includes classical distribution type, head-and-neck predominant type, prurigo type, and erythrodermic type. In addition, atypical manifestations, including elderly erythroderma, chronic polymorphic prurigo and palmoplantar eczema, are also recorded. Disease course is classified as persistent type, relapsing–remitting type, or adult-onset type. These classifications are incorporated to facilitate phenotype-guided analyses and precision medicine approaches in AD. To improve consistency across participating centers, clinical phenotype and disease course classifications will be assessed according to standardized operational definitions developed by the steering committee. Classification criteria will be shared with all participating institutions to promote uniform assessment practices.

### 2.4. Data Collection and Follow-Up

Baseline and longitudinal follow-up data include demographic characteristics, body mass index, smoking history, comorbidities, past and current treatment history, and physician-assessed disease severity. Physician-assessed outcomes include the Investigator’s Global Assessment (IGA) and Eczema Area and Severity Index (EASI). Patient-reported outcomes include the Patient Global Assessment (PaGA), itch Numeric Rating Scale (itch-NRS), Patient-Oriented Eczema Measure (POEM), and Dermatology Life Quality Index (DLQI). Pediatric versions of POEM and DLQI are used when appropriate.

Blood biomarkers include lactate dehydrogenase (LDH), thymus and activation-regulated chemokine (TARC), total immunoglobulin E (IgE), eosinophil ratio, and soluble interleukin-2 receptor (sIL-2R). These biomarkers are measured as part of routine clinical practice at participating institutions. TARC and sIL-2R are widely used standardized clinical laboratory tests in Japan and are assessed according to established laboratory procedures and reference ranges. Adverse events, treatment modifications, treatment discontinuation, and treatment-switch patterns are recorded throughout follow-up.

Patients are scheduled for follow-up approximately 1 month after enrollment and every 3 months thereafter, in accordance with the registry protocol. Assessments are performed during routine clinical practice, and the actual timing of visits may vary according to clinical circumstances. Longitudinal follow-up after treatment discontinuation is also encouraged and will focus on relapse patterns, delayed adverse events, remission duration, and treatment re-initiation using the same routine clinical assessments whenever feasible. Follow-up after treatment discontinuation will be performed during routine clinical visits whenever feasible. Although no dedicated digital health platform is currently implemented, participating investigators will encourage continued follow-up to facilitate the collection of post-discontinuation outcome data. A detailed overview of collected variables is provided in Table 1.

### 2.5. Data Management

Clinical data collected at each institution are anonymized using personal identification codes before transfer to the coordinating center. Correspondence tables linking patient identifiers and registry identification codes are securely maintained at each participating institution. Data are transferred through secure file-sharing systems and stored in password-protected databases at Kyushu University. Patient questionnaires may additionally be transferred using traceable secure postal services when necessary.

### 2.6. Outcomes

Primary Outcomes:

- Drug survival.

Secondary Outcomes:

- Improvement in disease severity;
- Incidence of adverse events;
- Associations between phenotype and treatment response;
- Associations between disease course and drug survival;
- Biomarker–treatment correlations;
- Treatment-switch patterns;
- Post-discontinuation disease course, relapse patterns, and treatment re-initiation.

### 2.7. Statistical Analysis

Continuous variables will be summarized as mean (SD) or median (IQR). Categorical variables will be expressed as frequency and percentage. Drug survival will be analyzed using the Kaplan–Meier method with log-rank testing. Multivariate Cox proportional hazards models will assess factors associated with treatment discontinuation, adjusting for phenotype, disease course, age, sex, and baseline severity. Linear or logistic regression models will evaluate predictors of clinical improvement. In comparative effectiveness analyses, propensity score-based methods may be used when appropriate to reduce confounding by indication and treatment selection bias. Analyses of drug survival, treatment sequences, and treatment-switching patterns may be performed using information on treatment initiation, discontinuation, switching, combination therapy, and re-initiation, as appropriate to the research question being addressed. Longitudinal analyses of biomarkers and clinical outcomes may be performed using mixed-effects models to account for repeated measurements over time. Phenotype–treatment interactions may be evaluated using interaction terms in multivariable models, and adjustment for multiple comparisons may be applied when appropriate. Missing data patterns will be evaluated, and missing data will be handled using appropriate statistical methods depending on the extent and pattern of missingness. Sensitivity analyses may be performed when appropriate. Appropriate adjustments for multiple comparisons will be applied in exploratory analyses when necessary.

### 2.8. Ethical Considerations

The study was approved by the Central Institutional Review Board of Kyushu University (Approval No. 25061). Written informed consent is obtained from all patients. Data are anonymized and securely managed.

## 3. Discussion

WJADR was designed to address the increasing complexity of systemic therapy for AD in the era of targeted therapeutics. WJADR was established based on the experience of the Western Japan Psoriasis Registry (WJPR). WJPR demonstrated the feasibility and clinical utility of multicenter dermatology registry studies in Japan through the evaluation of drug survival, safety, and infection risk analyses in psoriasis patients receiving systemic therapies [31,32,33]. WJADR extends this registry framework to AD, an inflammatory skin disease that shares chronic inflammatory characteristics with psoriasis but exhibits distinct immunological pathways and clinical phenotypes.

**Figure 2:**
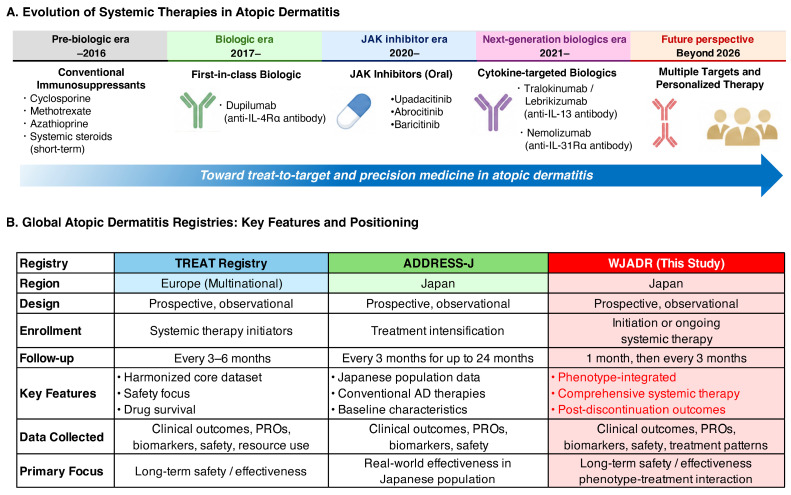
Global landscape of systemic therapies and real-world registries in atopic dermatitis. WJADR complements existing registries by integrating clinical phenotypes, biomarkers, and longitudinal outcomes including post-discontinuation courses in real-world practice. AD, atopic dermatitis; JAK, Janus kinase; IL, interleukin; Rα, receptor alpha.

As illustrated in Figure 2A, systemic therapy for AD has rapidly evolved from the era of conventional systemic immunosuppressive therapies to the biologic era and subsequently to the JAK inhibitor era. The expansion of therapeutic options has substantially improved disease control for patients with moderate-to-severe AD, while simultaneously generating new clinical challenges that are difficult to adequately address through conventional randomized controlled trials (RCTs) alone. These challenges include long-term safety monitoring, treatment sequencing, drug switching patterns, treatment persistence, and disease management after treatment discontinuation. In recent years, multiple targeted systemic therapies have become available for AD, including biologics targeting the IL-4/IL-13 pathway, selective IL-13 inhibitors, IL-31 receptor blockade, and JAK inhibitors. As a result, treatment selection and switching strategies have become increasingly complex in routine clinical practice. However, direct comparative evidence between these therapeutic options remains limited, and optimal treatment sequencing strategies have not been fully established. Consequently, real-world registries capable of evaluating long-term treatment persistence and treatment-switching patterns are becoming increasingly important.

While RCTs provide evidence of efficacy under controlled conditions, real-world registries are increasingly recognized as essential complements because they enable pharmacovigilance analyses, comparative effectiveness evaluations, and long-term assessment of treatment persistence in heterogeneous patient populations. The European TREAT registry emphasizes international data standardization and long-term safety assessment in systemic therapy for AD [18,19,20]. In contrast, WJADR incorporates a structured clinical phenotype classification system adapted to routine dermatology practice in Japan. This framework may contribute to phenotype-guided treatment optimization and prediction of long-term drug persistence. Furthermore, the registry structure may facilitate biomarker-integrated therapeutic strategy analyses. In particular, exploratory analyses may be able to determine whether specific phenotypes, such as prurigo-type AD or head-and-neck predominant AD, exhibit differential responsiveness or treatment persistence across systemic therapies. The Japanese ADDRESS-J registry primarily focuses on disease burden, patient-reported outcomes (PROs), and real-world treatment patterns in patients with moderate-to-severe AD [23]. Although ADDRESS-J significantly contributed to improving the understanding of AD burden in Japan, drug survival analyses, structured post-discontinuation follow-up, and phenotype–treatment interaction analyses were not central components of the registry design. In recent years, biologic-focused real-world cohort studies, including BioDay, PROSE, GLOBOSTAD, and BIOREP, have also been reported. These studies have provided important longitudinal data regarding the long-term effectiveness and safety of dupilumab-treated patients [24,25,26,27]. However, these studies primarily focused on biologic initiator cohorts and were not designed as comprehensive systemic therapy registries comparing multiple treatment strategies across heterogeneous AD phenotypes. In routine clinical practice, treatment modifications frequently occur because of inadequate response, adverse events, patient preferences, or comorbidities. Nevertheless, evidence regarding optimal treatment sequencing and switching strategies remains limited [16,34]. By longitudinally capturing treatment initiation, discontinuation, switching, and re-initiation, WJADR may provide valuable real-world insights into treatment pathways and help inform future therapeutic decision-making.

As summarized in Figure 2B, WJADR is positioned as a phenotype-integrated systemic therapy registry that combines drug survival analysis, longitudinal follow-up, and post-discontinuation outcome assessment within a unified real-world framework. By integrating clinical phenotypes, disease course classifications, biomarkers, and long-term treatment exposure, WJADR may provide a platform for precision medicine in AD. In addition, the relatively homogeneous Japanese population may complement predominantly European registry data by generating ethnicity-specific real-world evidence. Furthermore, WJADR includes both pediatric and adult patients. Although systemic treatment options for pediatric AD have expanded substantially in recent years, long-term real-world data regarding effectiveness, safety, and treatment persistence remain limited [16]. By enrolling patients across a broad age spectrum, WJADR may contribute to the generation of comprehensive real-world evidence spanning both pediatric and adult populations. Another notable feature of WJADR is the longitudinal collection of routinely measured biomarkers. Although TARC, total IgE, eosinophil ratio, and LDH are widely used in clinical practice in Japan, their ability to predict long-term treatment response, drug survival, and disease control remains incompletely understood. By integrating biomarker data with clinical phenotypes and treatment outcomes, WJADR may provide valuable insights into biomarker-guided treatment optimization and patient stratification in AD. In recent years, the management of AD has increasingly shifted from generalized inflammation control toward precision medicine approaches tailored to individual disease characteristics [17,30]. However, reliable predictors of treatment response remain limited, and selecting the most appropriate therapy for a given patient remains challenging. By integrating clinical phenotypes, disease course classifications, biomarkers, and treatment histories, WJADR may facilitate the development of predictive models and patient stratification strategies that support more personalized therapeutic decision-making.

Another important feature of WJADR is the incorporation of longitudinal follow-up after treatment discontinuation. Although long-term remission following biologic therapy has increasingly been reported, real-world evidence regarding relapse patterns, delayed adverse events, and treatment re-initiation strategies after treatment withdrawal remains limited [34,35,36,37]. WJADR may therefore provide clinically meaningful insights into long-term disease control beyond active treatment periods.

## 4. Conclusions

WJADR was designed as a phenotype-integrated real-world registry to address the increasing complexity of systemic therapy for AD in the era of targeted therapeutics. By integrating clinical phenotypes, biomarkers, drug survival, treatment-switch patterns, and post-discontinuation outcomes within a longitudinal framework, WJADR may provide a platform for precision medicine approaches in AD.

Several limitations should be acknowledged. As an observational registry, residual confounding and loss to follow-up cannot be completely eliminated, particularly for post-discontinuation follow-up conducted during routine clinical practice, and findings derived primarily from a Japanese population may not be fully generalizable to other ethnic groups.

Nevertheless, WJADR may serve as an important real-world evidence platform for promoting long-term safety evaluation, drug survival analysis, and phenotype-guided treatment optimization in systemic therapy for AD in Japan. Future integration with international registries and digital health technologies may further enhance the clinical utility of longitudinal AD registries.

## 5. Future Perspectives

As therapeutic options for AD continue to expand, longitudinal real-world registries are expected to play an increasingly important role in treatment optimization and precision medicine approaches. Future analyses using WJADR may facilitate biomarker-integrated treatment stratification and prediction of long-term treatment persistence across diverse clinical phenotypes. Furthermore, the integration of routinely available biomarkers, including TARC, total IgE, eosinophil ratio, and LDH, with clinical phenotypes may facilitate the development of practical prediction models for treatment response. In the future, such approaches may contribute to clinical decision-support tools capable of identifying patients most likely to benefit from specific biologics or JAK inhibitors. In addition, as the number of available systemic therapies continues to expand, defining optimal treatment sequencing strategies has become an important clinical challenge. Longitudinal data generated through WJADR may provide valuable insights into real-world treatment pathways, including treatment switching and re-initiation, thereby informing future evidence-based treatment strategies.

Another important future direction is the harmonization of Asian registry data with international registry initiatives such as TREAT. Such collaborative efforts may improve understanding of ethnicity-specific therapeutic responses and long-term safety profiles across different healthcare systems and patient populations.

Furthermore, advances in digital health technologies, electronic patient-reported outcomes, wearable devices, and artificial intelligence-based predictive modeling may further enhance the clinical utility of longitudinal AD registries. Integration of these approaches into real-world registry platforms may contribute to more personalized and data-driven management strategies for AD.

## Figures and Tables

**Table 1:** Core Data Elements Collected in the Western Japan Atopic Dermatitis Registry (WJADR).

Domain	Variables Collected
**Demographics**	Age, sex, body mass index (BMI), smoking status, alcohol use
**Clinical phenotype**	Typical (classical distribution, head-and-neck predominant, prurigo type, erythrodermic type); Atypical (elderly erythroderma, chronic prurigo, palmoplantar eczema)
**Disease course classification**	Persistent, relapsing-remitting, adult-onset
**Severity assessment**	IGA; EASI; Head and neck EASI
**Patient-reported outcomes (PROs)**	DLQI (adult and pediatric); POEM (adult and pediatric); Itch NRS; PaGA
**Biomarkers**	Peripheral blood biomarkers: WBC count; eosinophil percentage; total IgE; TARC; LDH; soluble IL-2 receptor
**Comorbidities**	Allergic comorbidities; cardiovascular diseases; metabolic disorders; psychiatric disorders; malignancy
**Infection screening**	Screening for HBV, HCV, HIV, HTLV-1, and tuberculosis
**Treatment exposure**	Conventional systemic therapy (cyclosporine); JAK inhibitors; Biologic agents (dupilumab, IL-13 inhibitors, nemolizumab); Phototherapy; Investigational therapies
**Adverse events**	Skin infections; herpes virus infections; pneumonia; tuberculosis; malignancy; cardiovascular events; ophthalmologic events
**Treatment history**	Start date; stop date; reason for discontinuation (inefficacy, adverse events, remission, economic reasons, etc.); switching patterns
**Longitudinal follow-up**	Scheduled follow-up assessments (approximately every 3 months)

Abbreviations: AD, atopic dermatitis; BMI, body mass index; DLQI, Dermatology Life Quality Index; EASI, Eczema Area and Severity Index; IGA, Investigator’s Global Assessment; JAK, Janus kinase; NRS, numeric rating scale; PaGA, Patient Global Assessment; POEM, Patient-Oriented Eczema Measure; TARC, thymus and activation-regulated chemokine.

## Data Availability

The data generated and analyzed in this study are not publicly available because the registry is ongoing and contains potentially identifiable clinical information. Data may be available from the corresponding author upon reasonable request and subject to approval by the relevant ethics committees and participating institutions.

## Abbreviations

AD	Atopic dermatitis
BMI	Body mass index
DLQI	Dermatology Life Quality Index
EASI	Eczema Area and Severity Index
IGA	Investigator’s Global Assessment
IgE	Immunoglobulin E
IQR	Interquartile range
JAK	Janus kinase
NRS	Numeric rating scale
PaGA	Patient Global Assessment
POEM	Patient-Oriented Eczema Measure
RCT	Randomized controlled trial
RWE	Real-world evidence
SD	Standard deviation
sIL-2R	Soluble interleukin-2 receptor
TARC	Thymus and activation-regulated chemokine
WJADR	Western Japan Atopic Dermatitis Registry
WJPR	Western Japan Psoriasis Registry
